# Supplementary material for: Activation of coagulation and proinflammatory pathways in thrombosis with thrombocytopenia syndrome and following COVID-19 vaccination
Source: Nat Commun. 2023 Oct 23;14:6703. doi: 10.1038/s41467-023-42559-x (PMC10593859; doi:10.1038/s41467-023-42559-x)
Supplement: Supplementary file 1 — Supplementary Information [file 41467_2023_42559_MOESM1_ESM.pdf]

Activation of Coagulation and Proinflammatory Pathways in Thrombosis with Thrombocytopenia Syndrome and Following COVID-19 Vaccination

First author: Malika Aid

Corresponding author: Dan Barouch

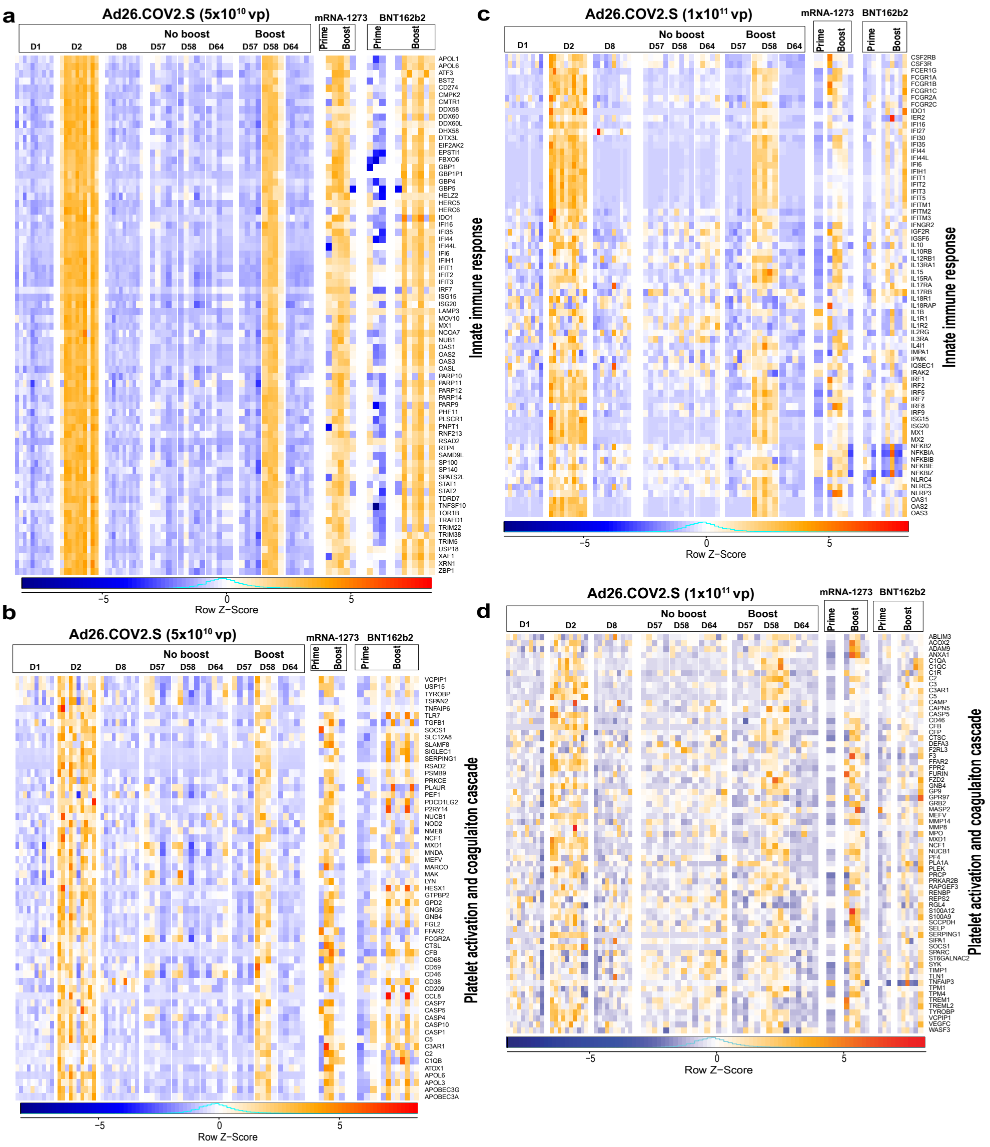

Supplementary Fig. 1. Transcriptomics changes following Ad26.COV2.S vaccination. **a-d**, Heatmaps showing the mRNA expression of markers of innate proinflammatory pathways (**a, c**) and coagulation cascade pathways (**b, d**) increased one day after initial immunization with 5x10^{10} vp or 1x10^{11} vp of Ad26.COV2.S and after the second immunization with Ad26.COV2.S and after mRNA-1273 and BNT162b2 boost immunization. The color gradient indicates increased (in red gradient) or decreased (in blue gradient) genes in blood.

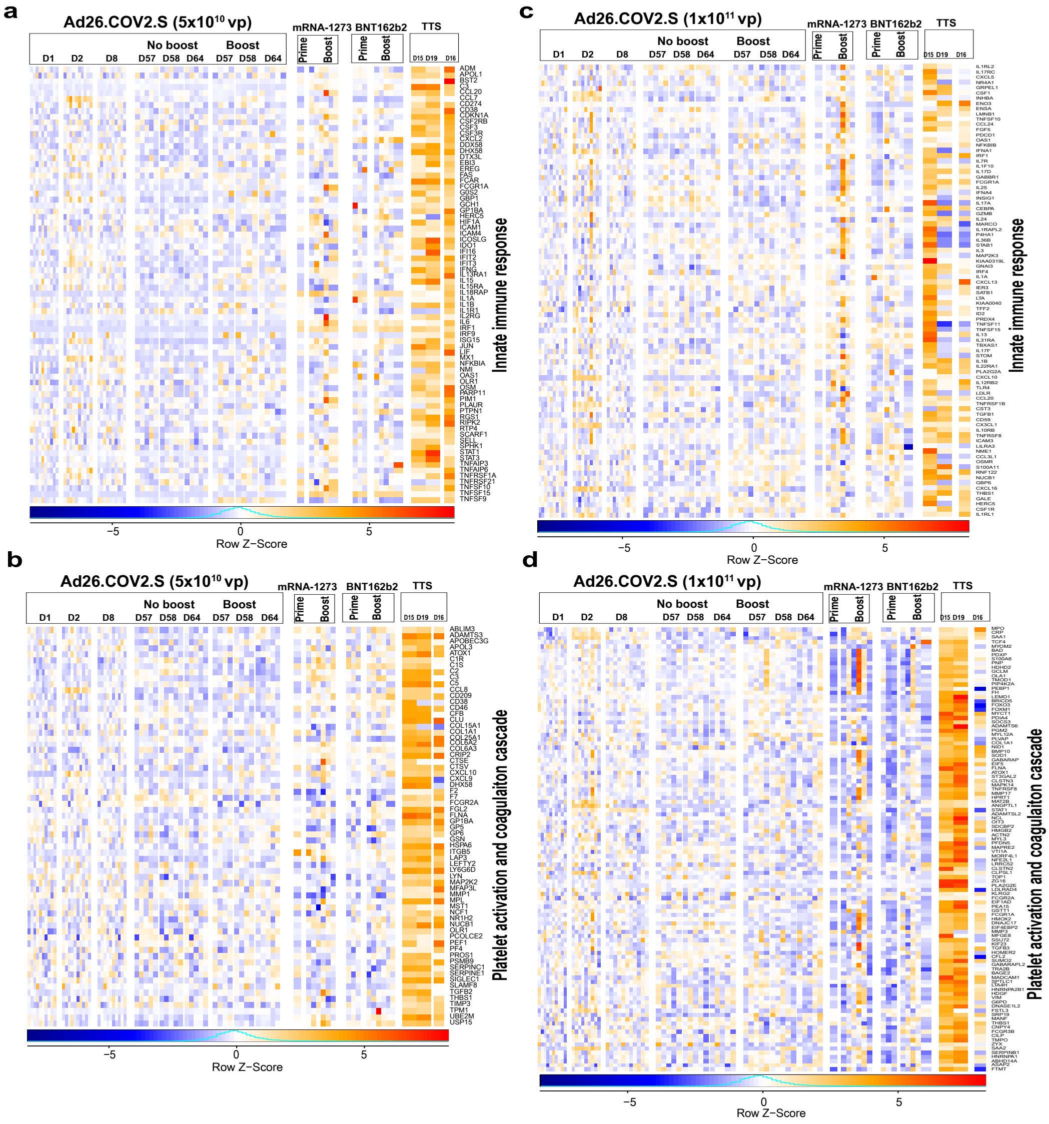

Supplementary Fig. 2. Proteomics changes after COVID-19 vaccines in healthy individuals and in TTS patients.

a-d, Heatmaps showing the serum levels of markers of innate proinflammatory pathways (**a, c**) and coagulation cascade pathways (**b, d**) increased one day after initial immunization with $5x10^{10}$ vp or $1x10^{11}$ vp of Ad26.COV2.S and after the second immunization with Ad26.COV2.S, after mRNA-1273 and BNT162b2 boost immunization and in TTS patients. The color gradient indicates increased (in red gradient) or decreased (in blue gradient) proteins in serum.

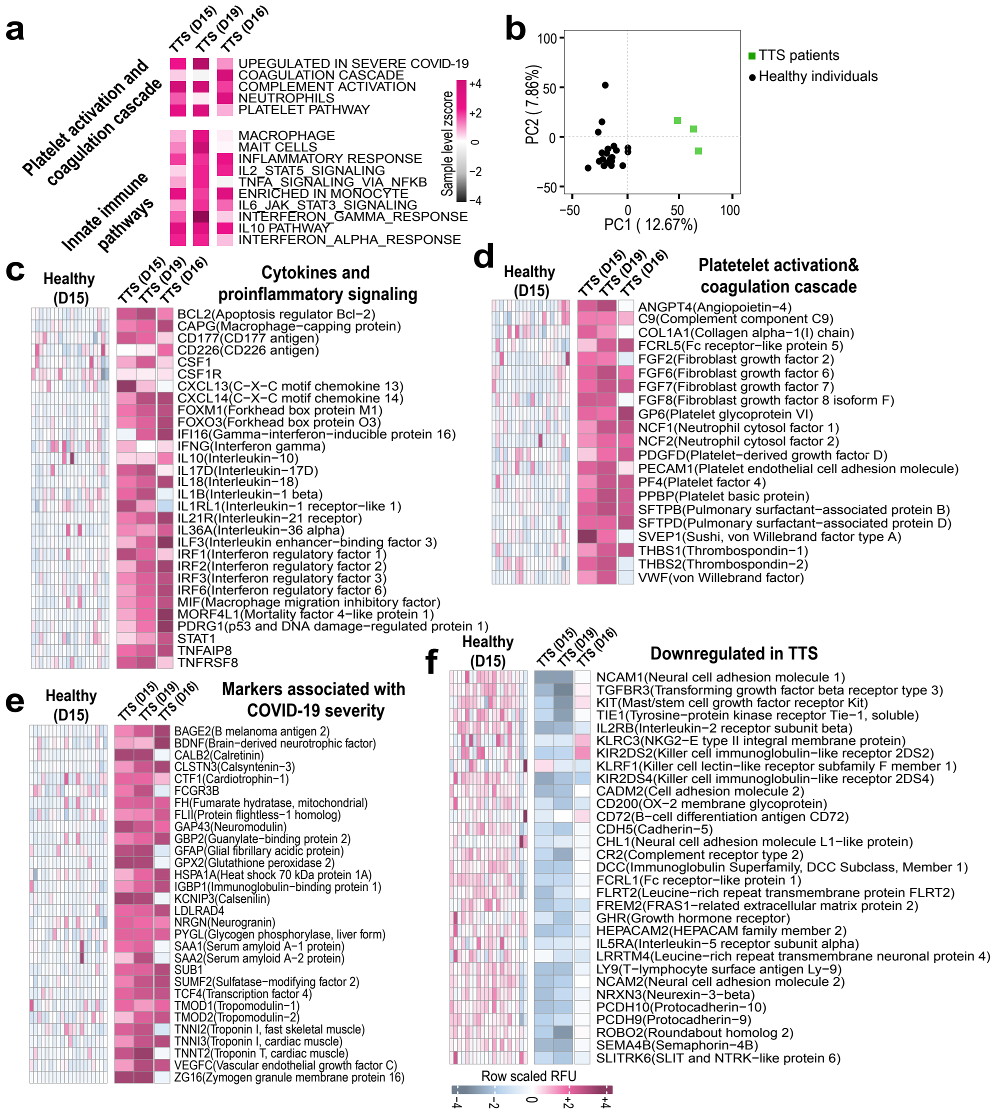

Supplementary Fig. 3. Persistent proinflammatory and coagulation cascades in TTS patients. a Heatmap showing the sample level score of innate pathways and coagulation cascade in plasma in TTS patients (n=2) on days 15, 16, and 19 after Ad26.COV2.S vaccination. **b** Principal component analysis (PCA) using 7000 proteins on days 15, 16, and 19 in TTS patients (n=2) in green squares and on day 15 in healthy vaccinated individuals (n=20) in dark circles, vaccinated with Ad26.COV2.S. **c-f** Plasma markers increased (c,d,e) or decreased (f) in TTS patients—color gradient ranging from dark blue for downregulated to dark pink for upregulated proteins. Comparison between TTS and healthy individuals was assessed using the limma t-test and BH-adjusted p<0.05. Source data are provided as a Source data file.

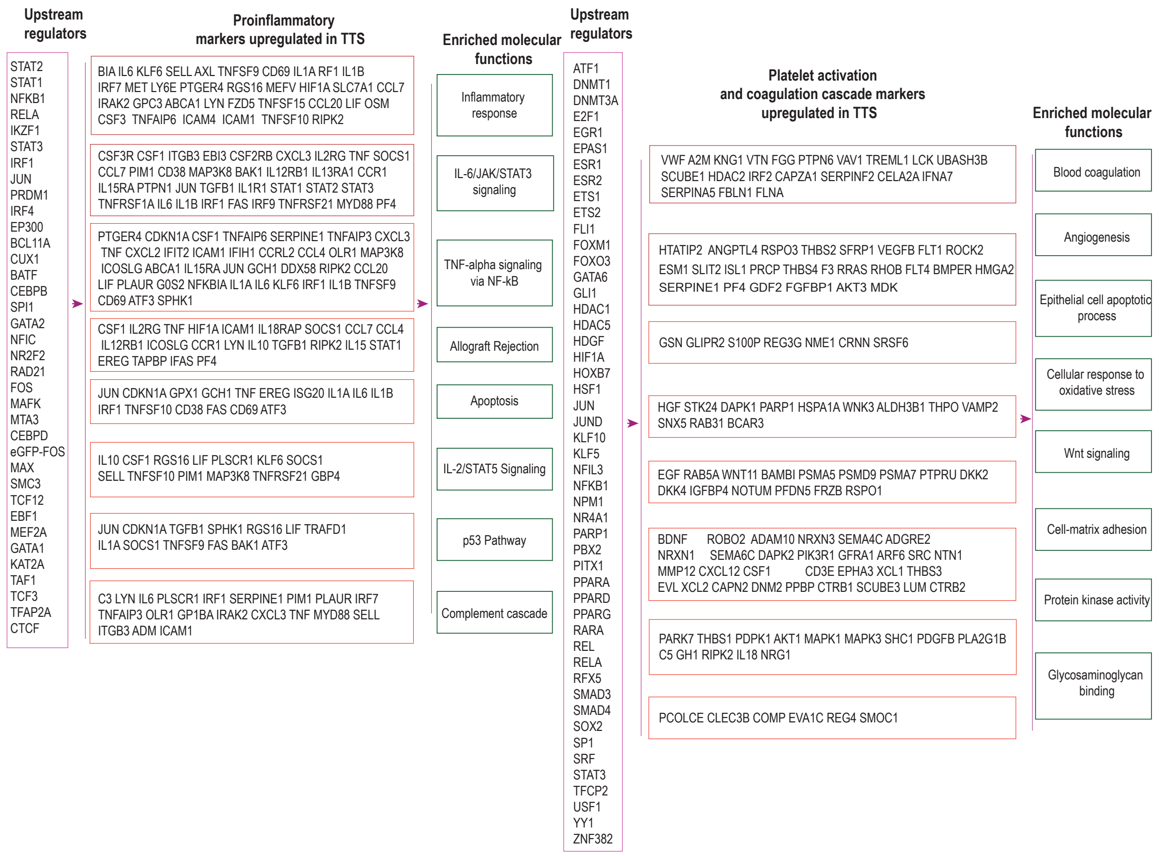

Supplementary Fig. 4. Upstream regulation analysis of pathways increased in TTS patients. Diagram showing the upstream regulators of proinflammatory markers and markers of the coagulation cascade increased in TTS patients regulated by major transcription factors associated with inflammation, epigenetics reprogramming, cell proliferation, apoptosis, angiogenesis, and coagulation cascade. Upstream regulation analysis was performed using EnrichR.

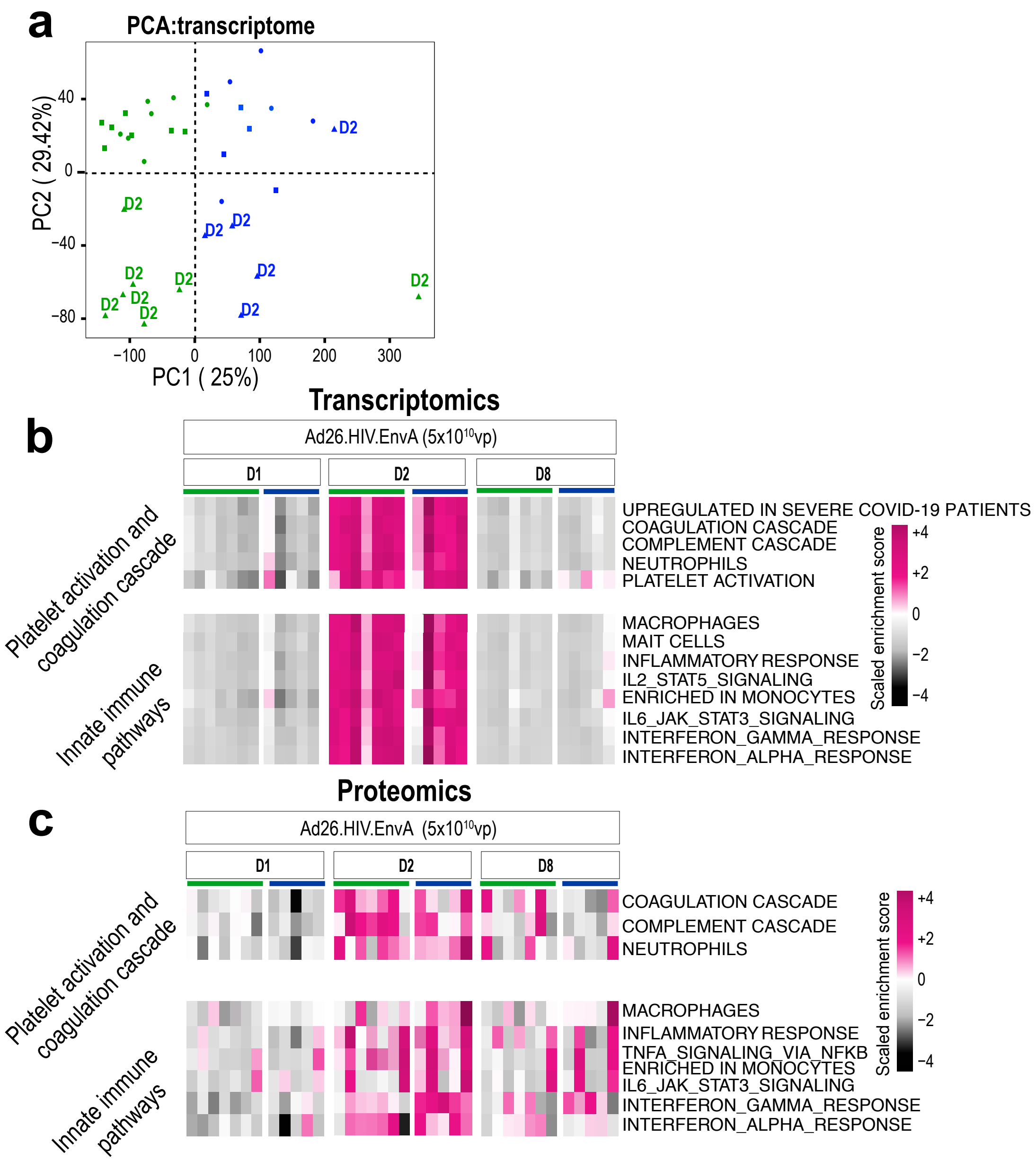

Supplementary Fig. 5. Transient activation of innate proinflammatory pathways and coagulation cascades following Ad26.HIV.EnvA vaccination. a PCA of peripheral blood transcriptomics at baseline (D1) and on day 1 (D2) following Ad26.HIV.EnvA vaccination in healthy individuals. Ad26.HIV seronegative (n=7) and seronegative (n=5) are shown in green and blue, respectively. **b and c**, Heatmaps of SLEA scores of innate immune pathways and coagulation cascades increased after one day (D2) by transcriptomics (**b**) and proteomics (**c**) following vaccination with Ad26.HIV.EnvA. These pathways largely resolved by one week (D8) following vaccination. Color gradient ranging from dark for unchanged to dark pink for upregulated genes and proteins. Source data are provided as a Source data file.

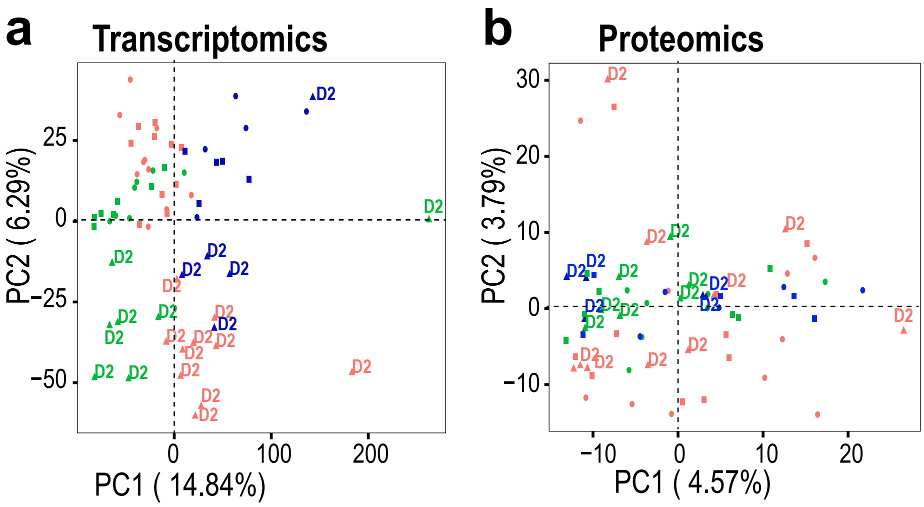

Supplementary Fig. 6. Common activation of innate proinflammatory pathways following Ad26.HIV.EnvA vaccination and Ad26.COV2.S vaccination in healthy individuals. a, b Principal component analysis (PCA) of peripheral blood transcriptomic (a) and serum proteomics (b) of Ad26.COV2.S, shown in red and Ad26.HIV.EnvA seronegative and seropositive, shown in green and blue, respectively, vaccinated healthy individuals at baseline (D1) and on days 1 (D2) and 7 (D8) following vaccination. Each dot represents an individual. D1: circle; D2: triangle and D8: square.

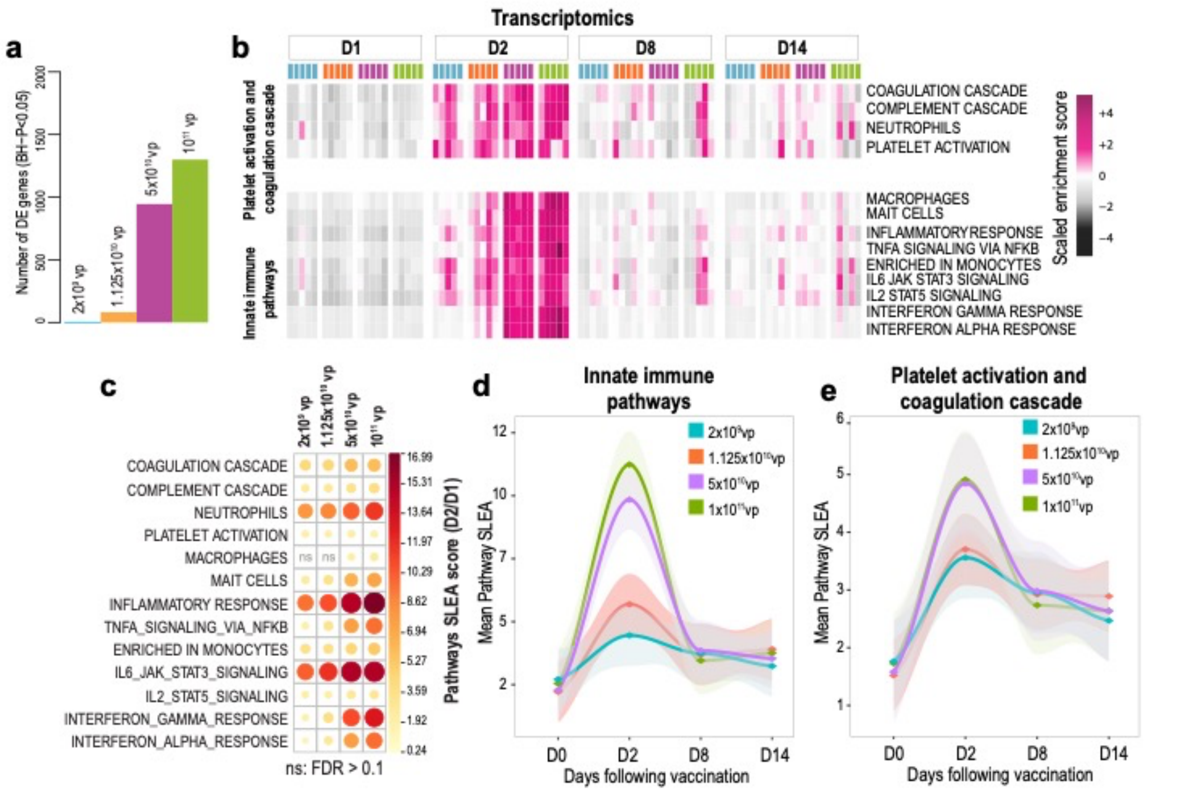

Supplementary Fig. 7. Reduced activation of coagulation and innate proinflammatory pathways with lower doses of Ad26.COV2.S in rhesus macaques.

a Barplots of the number of genes significantly modulated (Wald test, BH- adjusted p<0.05) on day 2 following immunization with various doses of Ad26.COV2.S in rhesus macaques.

b Heatmap of SLEA scores of innate immune pathways and coagulation cascades increased (GSEA: FDR< 0.10) after prime immunization (D2) with various doses of Ad26.COV2.S in rhesus macaques. Columns represent individual animals, and rows represent pathways. Color gradient ranging from dark for decreased to dark pink for upregulated genes.

c, Circle plot represents the pathways SLEA score ratio of D2/D1. Circle size corresponds to the D2/D1 SLEA ratio for each vaccine dose. **d**,**e** Plots of mean SLEA scores of innate immune and coagulation cascade pathways across all animals showing a dose-dependent activation of these pathways that resolved after one week following Ad26.COV2.S immunization. Source data are included in the Source data file.

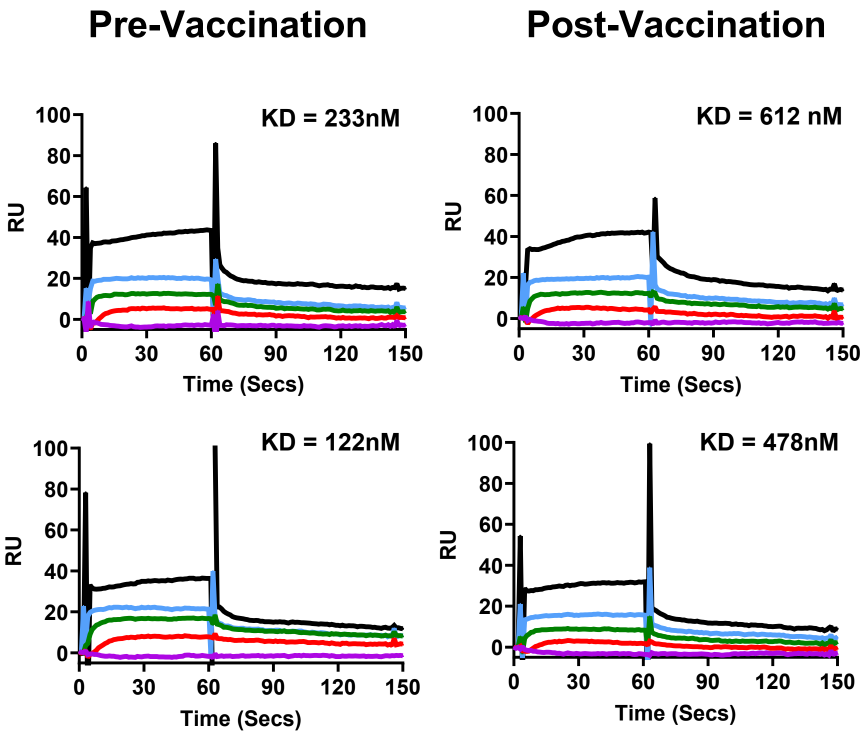

Supplementary Fig. 8. Ad26 binding to platelet factor 4 (PF4).

Partial inhibition of Ad26 binding to PF4 by surface plasmon resonance by serum following Ad26.COV2.S vaccination compared with serum prior to vaccination. Ad26 was immobilized on a C1 sensor chip, 1:5 pre-diluted polyclonal pre-Ad26 or post-Ad26 vaccination human serum flowed over the bound Ad26 followed by a titration of recombinant human platelet factor 4 (PF-4). PF-4 concentrations in sensorgrams are 3000nM (black) 1000nM (blue), 333nM (green), 111nM (red), and 37nM (purple).

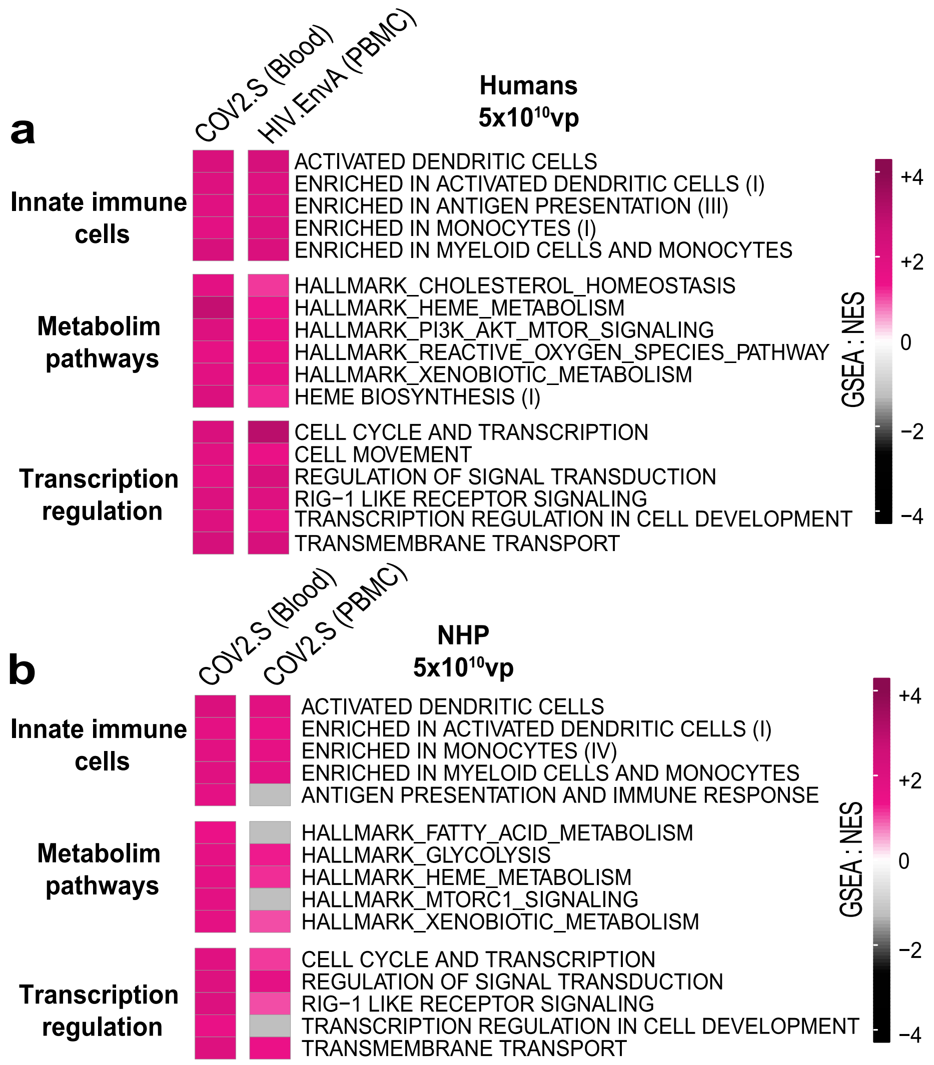

Supplementary Fig. 9. Upregulation of innate immune cell activation pathways, metabolism, and cell cycle one day following Ad26.COV2.S and Ad26.HIV.EnvA vaccination in healthy individuals and rhesus macaques.

a, b, Heatmaps of the GSEA normalized score of pathways of innate immune cell activation, metabolism, and cell cycle significantly increased on day 1 following immunization (FDR q value <0.10) in healthy individuals vaccinated with Ad26.COV2.S or Ad26.HIV.EnvA (a) and in rhesus macaques vaccinated with Ad26.COV2.S (b). Color gradient ranging from dark for decreased to dark pink for upregulated genes.
