# Supplementary material for: Activation of coagulation and proinflammatory pathways in thrombosis with thrombocytopenia syndrome and following COVID-19 vaccination
Source: Nat Commun. 2023 Oct 23;14:6703. doi: 10.1038/s41467-023-42559-x (PMC10593859; doi:10.1038/s41467-023-42559-x)
Supplement: Supplementary file 3 — Description of Additional Supplementary Files [file 41467_2023_42559_MOESM3_ESM.pdf]

Description of Additional Supplementary Files

Supplementary Data 1. List of proteins measured in serum using the SomaScan assay in TTS patients (n=2), where column 1 shows the protein name, column 2: the protein Z-score, and column 3: the protein/gene NCBI link.

Supplementary Data 2. Transcriptomic and proteomics signatures increased on D2 (prime) compared with D58 (boost) immunization with Ad26.COV2.S (5x10^10vp). Pathway GSEA normalized score (NES) and the fold discovery rate (FDR q value) were shown for each pathway.
