# Supplementary material for: Activation of coagulation and proinflammatory pathways in thrombosis with thrombocytopenia syndrome and following COVID-19 vaccination
Source: Nat Commun. 2023 Oct 23;14:6703. doi: 10.1038/s41467-023-42559-x (PMC10593859; doi:10.1038/s41467-023-42559-x)
Supplement: Supplementary file 6 — Reporting Summary [file 41467_2023_42559_MOESM6_ESM.pdf]

nature portfolio

Corresponding author(s): Dan Barouch
Last updated by author(s): Sep 30, 2023

Reporting Summary

Nature Portfolio wishes to improve the reproducibility of the work that we publish. This form provides structure for consistency and transparency in reporting. For further information on Nature Portfolio policies, see our Editorial Policies and the Editorial Policy Checklist.

Statistics

For all statistical analyses, confirm that the following items are present in the figure legend, table legend, main text, or Methods section.

n/a	Confirmed	
☐	☒	The exact sample size (*n*) for each experimental group/condition, given as a discrete number and unit of measurement
☐	☒	A statement on whether measurements were taken from distinct samples or whether the same sample was measured repeatedly
☐	☒	The statistical test(s) used AND whether they are one- or two-sided<br>*Only common tests should be described solely by name; describe more complex techniques in the Methods section.*
☐	☒	A description of all covariates tested
☐	☒	A description of any assumptions or corrections, such as tests of normality and adjustment for multiple comparisons
☐	☒	A full description of the statistical parameters including central tendency (e.g. means) or other basic estimates (e.g. regression coefficient) AND variation (e.g. standard deviation) or associated estimates of uncertainty (e.g. confidence intervals)
☐	☒	For null hypothesis testing, the test statistic (e.g. *F*, *t*, *r*) with confidence intervals, effect sizes, degrees of freedom and *P* value noted<br>*Give P values as exact values whenever suitable.*
☒	☐	For Bayesian analysis, information on the choice of priors and Markov chain Monte Carlo settings
☒	☐	For hierarchical and complex designs, identification of the appropriate level for tests and full reporting of outcomes
☒	☐	Estimates of effect sizes (e.g. Cohen's *d*, Pearson's *r*), indicating how they were calculated

Our web collection on statistics for biologists contains articles on many of the points above.

Software and code

Policy information about availability of computer code

Data collection	No software was used for data collection
Data analysis	Bulk RNA sequencing data were aligned to reference genomes using STAR version 2.7.3a; normalization and differential gene expression were performed using DESeq2_1.36.0. Proteomics data analysis was performed using the limma 3.41.15 R package. Gene set enrichment analysis was performed using GSEA GSEA 4.2.3 software. All the figures were generated using R studio 2022.02.0 Build 443. Comparisons between groups were performed using GraphPad Prism Software version 9.4.0 .

For manuscripts utilizing custom algorithms or software that are central to the research but not yet described in published literature, software must be made available to editors and reviewers. We strongly encourage code deposition in a community repository (e.g. GitHub). See the Nature Portfolio guidelines for submitting code & software for further information.

Data

Policy information about availability of data

All manuscripts must include a data availability statement. This statement should provide the following information, where applicable:

- Accession codes, unique identifiers, or web links for publicly available datasets
- A description of any restrictions on data availability
- For clinical datasets or third party data, please ensure that the statement adheres to our policy

The data discussed in this publication have been deposited in NCBI's Gene Expression Omnibus and are accessible through GEO Series accession number GSE220659 (https://www.ncbi.nlm.nih.gov/geo/query/acc.cgi?acc= GSE220659).

Human research participants

Policy information about studies involving human research participants and Sex and Gender in Research.

Reporting on sex and gender	The study is not powered to analyze by sex and gender
Population characteristics	Eligible participants were 18 to 55 years old (inclusive) and negative for SARS-CoV-2 infection by screening nasopharyngeal polymerase chain reaction and serum immunoglobulin testing. Eligible participants also had a body mass index of 30 or less (calculated as weight in kilograms divided by height in meters squared); were healthy, in the investigator's clinical judgment, as confirmed by medical history, physical examination, clinical laboratory assessments, and vital signs performed at screening; and did not have comorbidities related to an increased risk of severe COVID-19.
Recruitment	All participants gave written informed consent and successfully completed an assessment of understanding before the initiation of study procedures. Twenty-five participants were randomized to receive 1 or 2 intramuscular injections with 5× 10^10 viral particles or 1×10^11 viral particles of Ad26.COV2.S vaccine or placebo administered on day 1 and day 57 (5 participants in each group).
Ethics oversight	Beth Israel Deaconess Medical Center Institutional Review Board.

Note that full information on the approval of the study protocol must also be provided in the manuscript.

Field-specific reporting

Please select the one below that is the best fit for your research. If you are not sure, read the appropriate sections before making your selection.

- [x] Life sciences
- [ ] Behavioural & social sciences
- [ ] Ecological, evolutionary & environmental sciences

For a reference copy of the document with all sections, see nature.com/documents/nr-reporting-summary-flat.pdf

Life sciences study design

All studies must disclose on these points even when the disclosure is negative.

Sample size	No statistical analysis was used to predetermine sample size. Study sample size was determinant based on available samples.
Data exclusions	No data were excluded from the analysis
Replication	We performed studies in rhesus macaques with similar results.
Randomization	Samples were allocated randomly into experimental groups
Blinding	Investigators were blinded to group allocation

Reporting for specific materials, systems and methods

We require information from authors about some types of materials, experimental systems and methods used in many studies. Here, indicate whether each material, system or method listed is relevant to your study. If you are not sure if a list item applies to your research, read the appropriate section before selecting a response.

Materials & experimental systems

n/a	Involved in the study
☒	☐ Antibodies
☒	☐ Eukaryotic cell lines
☒	☐ Palaeontology and archaeology
☐	☒ Animals and other organisms
☐	☒ Clinical data
☒	☐ Dual use research of concern

Methods

n/a	Involved in the study
☒	☐ ChIP-seq
☒	☐ Flow cytometry
☒	☐ MRI-based neuroimaging

Animals and other research organisms

Policy information about studies involving animals; ARRIVE guidelines recommended for reporting animal research, and Sex and Gender in Research

Laboratory animals	30 outbred Indian-origin adult 5-8 kg rhesus macaques (Macaca mulatta)
Wild animals	This study did not involve wild animals
Reporting on sex	Indian-origin adult male (10) and female (20) rhesus macaques (Macaca mulatta) were randomly allocated to groups.
Field-collected samples	All animals were housed at Bioqual, Inc. (Rockville, MD).
Ethics oversight	Animal studies were conducted in compliance with all relevant local, state, and federal regulations and were approved by the Bioqual Institutional Animal Care and Use Committee (IACUC).

Note that full information on the approval of the study protocol must also be provided in the manuscript.

Clinical data

Policy information about clinical studies

All manuscripts should comply with the ICMJEguidelines for publication of clinical research and a completedCONSORT checklist must be included with all submissions.

Clinical trial registration	NCT04436276 ; NCT01103687.
Study protocol	https://clinicaltrials.gov/ct2/show/NCT04436276 ; https://clinicaltrials.gov/ct2/show/NCT01103687
Data collection	NCT04436276: Twenty-five participants were enrolled from July 29, 2020, to August 7, 2020, and the follow-up for this day 71 interim analysis was completed on October 3, 2020; follow-up to assess durability will continue for 2 years. This study was conducted at a single clinical site in Boston, Massachusetts, as part of a randomized, double-blind, placebo-controlled phase 1 clinical trial of Ad26.COV2.S.<br><br>NCT01103687: This study enrolled healthy, HIV-uninfected people. 16 Ad26– and 8 Ad26+ individuals. Participants were randomly assigned to receive either the rAd26 vaccine or a placebo vaccine. All vaccines were injected into the upper arm. At the vaccination study visit, participants underwent a medical and medication history review, physical examination, and a rectal sampling procedure.
Outcomes	NCT04436276: In this phase 1 study, a single immunization with Ad26.COV2.S induced rapid binding and neutralization antibody responses as well as cellular immune responses.<br><br>NCT01103687: In this phase 1 study, Ad26-vectored HIV-1 Env vaccine elicited both systemic and mucosal immune responses in humans.
